# A Risk Assessment and Planning Tool to Prevent Sudden Unexpected Death in Infancy: Development and Evaluation of The Baby Sleep Planner

**DOI:** 10.2196/49952

**Published:** 2024-02-22

**Authors:** Anna Pease, Jenny Ingram, Becky Lambert, Karen Patrick, Kieren Pitts, Peter J Fleming, Peter S Blair, The Baby Sleep Project Family Advisory Group

**Affiliations:** 1 Population Health Sciences Bristol Medical School University of Bristol Bristol United Kingdom; 2 Royal United Hospitals Bath NHS Foundation Trust Bath United Kingdom; 3 Research IT University of Bristol Bristol United Kingdom

**Keywords:** safer sleep, parent education, co-design, process evaluation, sudden infant death syndrome, SIDS, sleep, baby, babies, infant, infants, prototype, interface, develop, development, sleeping, pattern, tool, parent, infant mortality, risk, risks, assessment, death, mortality, parents, parenting, risk assessment, sudden unexpected death in infancy, SUDI, approach, antenatal, postnatal, user testing, user experience, web-based, experience, experiences, attitude, attitudes, opinion, perception, perceptions, perspective, perspectives

## Abstract

**Background:**

Successful national safer sleep campaigns in the United Kingdom have lowered the death rates from sudden unexpected death in infancy (SUDI) over the past 3 decades, but deaths persist in socioeconomically deprived families. The circumstances of current deaths suggest that improvements in support for some families to follow safer sleep advice more consistently could save lives.

**Objective:**

This study aimed to develop and evaluate a risk assessment and planning tool designed to improve the uptake of safer sleep advice in families with infants at increased risk of SUDI.

**Methods:**

A co-design approach was used to develop the prototype interface of a web-based tool with 2 parts: an individual SUDI risk assessment at birth and a downloadable plan for safety during times of disruption. The advice contained within the tool is concordant with national guidance from the Lullaby Trust, the United Nations International Children’s Emergency Fund (UNICEF), and the National Institute for Health and Care Excellence. User testing of the prototype tool was conducted by inviting health visitors, midwives, and family nurses to use it with families eligible for additional support. Qualitative interviews with health professionals and families allowed for iterative changes to the tool and for insights into its function and influence on parental behavior.

**Results:**

A total of 22 health professionals were enrolled in the study, of whom 20 (91%) were interviewed. They reported appreciating the functionality of the tool, which allowed them to identify at-risk families for further support. They felt that the tool improved how they communicated about risks with families. They suggested expanding its use to include relevance in the antenatal period and having versions available in languages other than English. They reported using the tool with 58 families; 20 parents gave consent to be interviewed by the research team about their experiences with the tool. Families were positive about the tool, appreciated the trustworthy information, and felt that it was useful and appropriate and that the plans for specific infant sleeps would be of benefit to them and other family members.

**Conclusions:**

Our tool combines risk assessment and safety planning, both of which have the potential to improve the uptake of lifesaving advice. Refinements to the tool based on these findings have ensured that the tool is ready for further evaluation in a larger study before being rolled out to families with infants at increased risk.

## Introduction

### Background

Recent data from the National Child Mortality Database show a strong link between known risks in the sleep environment (eg, infant prone sleeping and hazardous cosleeping) and sudden unexpected death in infancy (SUDI) in 2020, with at least 1 known factor present in 75% of the deaths [1]. These data also show the scale of inequalities, with a significantly larger proportion of unexplained deaths of infants living in the most deprived neighborhoods (42%) than of those in the least deprived neighborhoods (8%), a 5-fold increase. In 2017, a consensus process (based on the James Lind Alliance Priority Setting model) in identifying research priorities to reduce SUDI rated “developing and evaluating new ways to make safe sleep interventions more effective” as the top priority in the United Kingdom [2]. More recently, the Child Safeguarding Practice Review Panel has called for further efforts to increase the uptake of safer sleep advice in families in which the risks of SUDI are much higher than in the general population [3]. The Baby Sleep Planner was designed in response to recommendations to target support and resources to those families with infants most at risk, provide tailored and personalized risk information, and facilitate planning for infant safety during times when the normal routine is disrupted [3]. Risk assessment calculators for SUDI at the time of birth have not been widely used before in the United Kingdom, but the shift to increased prevalence among families living in the most deprived neighborhoods makes this more viable. The tool comprises 2 parts: a risk assessment at birth showing infant risk based on background and neonatal characteristics and a sleep environment planning section that provides an individualized plan for safety that can be downloaded as an image and shared with family and friends. Currently, most safer sleep advice and guidance in the United Kingdom is given by midwives, health visitors, and specialist nurses. Message delivery is often compounded by limited time and conflicting advice from multiple sources [4]. Health professional resources aim to increase parental knowledge of SUDI risks, and recent qualitative interviews with them suggest that they would welcome a targeted approach for families with infants at the greatest risk using parental input to come up with realistic strategies during disrupted routines [3,5]. A recent review of interventions to increase the uptake of safer sleep advice in families of infants at increased risk concluded that approaches moving away from “information giving” toward “information exchange” may be more effective for this group [6]. Using the detailed evidence we collected in Bristol and working closely with families whose infants are at higher risk to understand parental decision-making, we had a unique opportunity to derive a targeted intervention [4,7].

### Objectives

This paper describes the development and evaluation of a web-based tool that aimed to improve the uptake of safer sleep advice in families with infants at increased risk of SUDI. The Baby Sleep Planner was designed together with health professionals, families, other academics, and a team of software developers. The objectives of this study were as follows:

1. To use a co-design approach to develop a prototype web-based interface that the target group can use
2. To conduct user testing of the tool, including training and data capture of tool answers
3. To conduct qualitative interviews with health professionals and family members who have used the tool to understand how the tool works in real-world conditions and refine it for testing in a future study

### Theory-Based Approach

The Medical Research Council’s guidance on the development and evaluation of complex interventions puts developing and testing theory as a core concept [8]. By using previous research on the influences on behavior of our priority group, we hope to provide a transparent theoretical underpinning that can be tested in a future study.

The risk assessment and planning tool is based on a Capability, Opportunity, and Motivation–Behavior (COM-B; behavior change) model that considers the sources of behavior along with the behavior change techniques likely to work on the target behaviors [9]. The COM-B model proposes that capability, opportunity, and motivation interact to predict behavior and that intervention designers should consider how to influence these constructs. Our previous studies have provided the basis for identifying the behavioral targets for intervention and their corresponding behavior change techniques [4,5,10,11]. The goal of our intervention is to enable parents with infants at most risk of SUDI to consistently provide a safe sleep environment for their infants, especially during disrupted routines. We chose techniques that focus on increasing *capability* by providing information about their baby’s risk; *opportunity* by using their environmental context and resources to develop realistic strategies for providing a safe sleeping environment; and *motivation* through planning, goal setting, and increasing confidence (Table 1).

**Table 1:** Model of the intervention showing the Capability, Opportunity, and Motivation–Behavior (COM-B) model using Theoretical Domains Framework (TDF) domains and corresponding behavior change techniques.

COM-B construct and subconstruct		TDF domain	Finding or problem	Corresponding behavior change technique	Proposed mechanism of action of the Baby Sleep Planner
**Capability**					
	Physical capability	Physical skills	Advice interpreted differently or misunderstood	Instruction on how to perform the behavior	Increased confidence to provide a safer sleep environment
	Psychological capability	Knowledge Cognitive and interpersonal skills; memory, attention, and decision processes Behavioral regulation	Safer sleep advice too generic and not individualized Disruption to the routine can create unplanned risky situations	Information about health consequences Behavior substitution	Increased understanding of their own infant’s risk status Prioritizes safety over convenience
**Opportunity**					
	Physical opportunity	Environmental context and resources	Poor-quality accommodation makes following advice harder	Restructure the physical environment Reduce exposure to cues for the behavior	Increased confidence to maintain safety in nonstandard situations
	Social opportunity	Social influences	Burden of following advice loaded on primary carer or mother	Social support	Sharing the plans with wider family and friends reduces burden and increases safety when the infant is cared for by others
**Motivation**					
	Reflective motivation	Social or professional role and identity Beliefs about capabilities; optimism Beliefs about consequences; intentions Goals	Trusted sources provide impactful information “Just this once” mentality puts infants at increased risk during times of disruption	Credible source Goal setting Behavioral contract	Health professionals become trusted, and advice increases in credibility Increased confidence to follow a personalized plan for safety
	Automatic motivation	Reinforcement Emotion	Fear of SUDI^a^ can be stressful and overwhelming	Reduce negative emotions	Increased confidence that the plan is achievable and realistic

^a^SUDI: sudden unexpected death in infancy.

## Methods

### Ethical Considerations

The full study protocol was reviewed and given a favorable ethical opinion by the London – Chelsea Research Ethics Committee and granted Health Research Authority approval on June 21, 2022 (reference 22/PR/0445). Interview participants were compensated for their time with shopping vouchers.

### Professional Advisory Group

A group of experts was consulted to make sure that the content and advice within the tool supported the national advice for safer sleep. These experts comprised a professor of neonatology; a professor of midwifery and nursing; a professor of anthropology; a chief executive of a national SUDI charity; and a specialist health visitor for Gypsy, Roma, and Traveler families. Their input was sought during the development of the tool in conjunction with our co-design meetings.

### Co-Design Meetings

**Figure 1:**
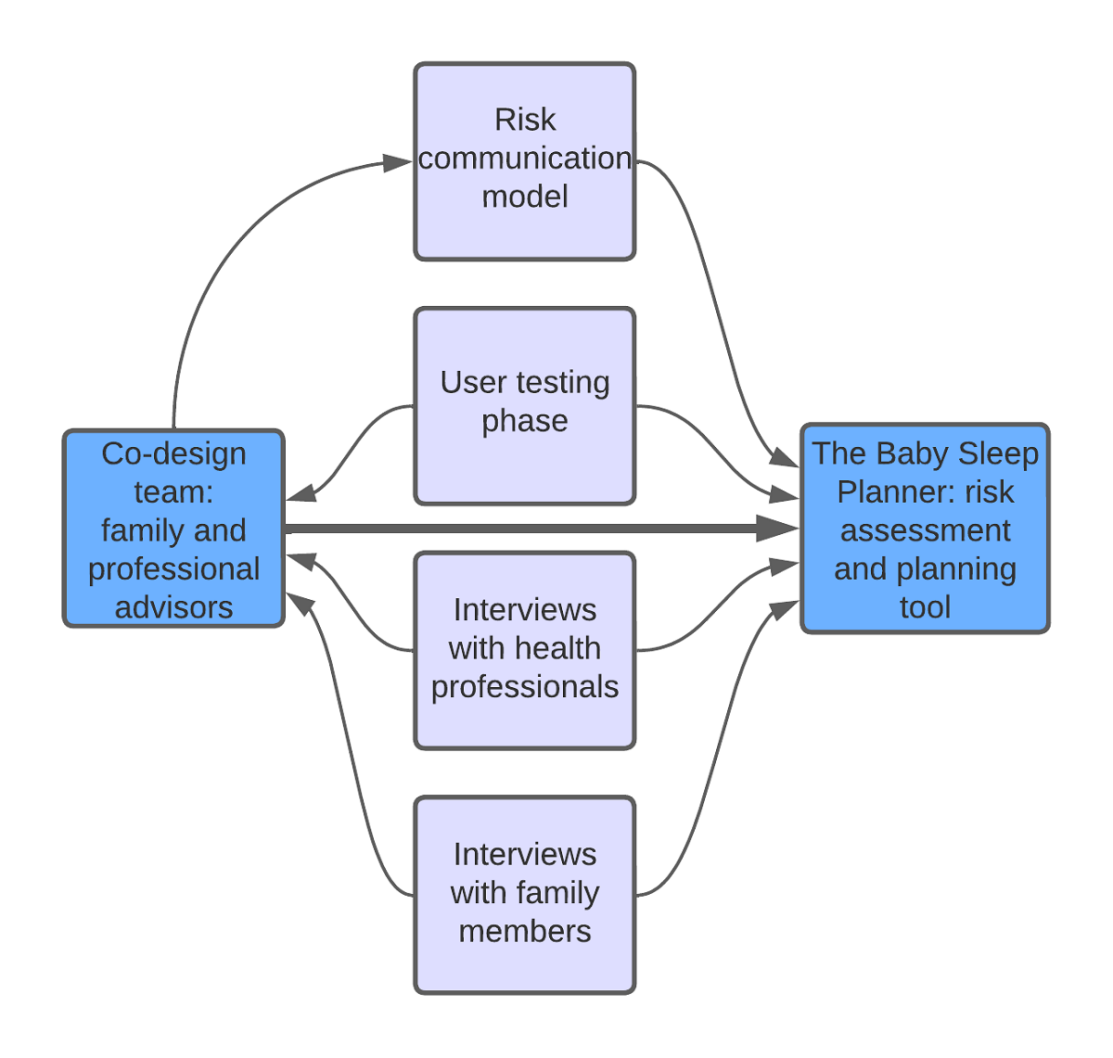
Co-design process showing data sources used to design the risk assessment and planning tool.

Before developing the tool, we engaged a family advisory group made up of 15 families with infants at risk of SUDI or who were affected by SUDI. The group met regularly both before and during the evaluation phase to influence the concept and design of the tool. Members of this group were invited to join via local health visitors, family nurses, and our study website and social media accounts as well as through Little Lullaby, a branch of the Lullaby Trust specifically for young parents. Figure 1 shows the overall process of tool design, including the influence of the co-design team and evaluation activities.

### Health Professional Recruitment

A total of 3 health professional roles were included in our process evaluation of the tool: midwives, health visitors, and Family Nurse Partnership (FNP) nurses. The midwives were all from a single community-based team for vulnerable and high-risk families. FNP nurses work solely with mothers aged ≤19 years, and 1 team from Bristol was invited to take part. Health visitors working with vulnerable families in 3 local areas (Bristol, North Somerset, and South Gloucestershire) were asked to volunteer to take part in the study by their managers. Study information sheets detailed all aspects of the research and included information on data security. Written consent to use the tool and take part in an interview was collected before participating in the study. All data collection for the evaluation followed the UK policy framework for health and social care research [12], including adhering to strict data protection guidance. Data were stored on secure university servers only accessible to members of the study team.

### Health Professional Training

A training package comprising a handbook, video presentation, Microsoft PowerPoint (Microsoft Corp) slide show, and 30-minute session with a member of the study team was provided to each health professional. The handbook included information on the background of the tool, the evidence base, how it was developed, the structure of the tool, and details about how to use it with families. The video presentation covered both the structure and use of the tool and was presented at a 30-minute training session attended by every health professional. Completion of the training was a prerequisite for being sent the link to the tool. Health professionals were supported throughout the study with dedicated email and phone contact.

### Health Professional Interviews

Semistructured interviews with health professionals provided insights at each stage into the conditions of delivery, including *adoption* of the tool (how it was used, which resources were used, how families were chosen, and which family members engaged), *appropriateness and acceptability* (response from professionals and ease of use), and *fidelity* (the details of implementation into practice vs what we envisaged). Health professionals were also asked about scope for widening the tool beyond safer sleep, for their suggestions for how to do this, and which other infant health or well-being topics would be relevant to their work with families experiencing poverty. The interview topic guide was developed with input from our professional advisors, and iterations were made as the interviews progressed.

### Family Interview Recruitment

Consent to be shown the tool was given verbally to the health professionals during initial conversations on safe sleep. Separate consent was also embedded into the tool to allow researchers to view the responses. Thus, it was possible to consent to be shown the tool without collecting any data or participating in an interview. Consent to be contacted regarding a possible interview about their experience using the tool was passed on to the research team via the health professional for follow-up. A member of the research team contacted each family member with a study information sheet and consent form. Recruitment took place via telephone, email, or SMS text message depending on participant preference.

### User Testing (Health Professionals Using the Tool With Families)

The link to the Baby Sleep Planner was provided for a period of 12 weeks to allow enough time for each health professional to use the tool with 5 to 6 families. Once health professionals had recruited enough families, they took part in a qualitative interview. At the end of the user testing phase, the data were downloaded. Where consent was given, the tool collected data on each answer to the risk assessment and planning sections and which plan options were chosen. All questions were multiple choice, no personal details could be entered into the tool, and no responses were stored locally on any device to prevent accidental data breaches or identification of any participants.

### Family Interviews

Qualitative telephone interviews used a topic guide with families focused on *acceptability* (engagement with the tool and ease of use), *appropriateness* (language and literacy access and perceived targeting by professionals), and evidence of *influence on behavior* (experiences with using the plan and spreading awareness to other parents or carers). The interview topic guide was developed with input from our family advisory group, with iterations as the interviews progressed. Individuals aged <16 years, anyone who lacked the cognitive capacity to consent, and anyone unable to complete an interview in English were not eligible to take part in the study.

### Interview Analysis

The interviews took place via telephone or face-to-face. The audio recordings were transcribed, anonymized, coded, and investigated using a framework analysis allowing for a systematic approach to generating themes [13]. An initial analytical framework of codes was developed inductively using the first 5 transcripts, agreed upon by 3 team members, and then applied consistently (deductively) across the remaining transcripts. Separate frameworks were developed for family and health professional interviews. Team members coded transcripts using double coding across 50%, and discrepancies were resolved through team discussion.

## Results

### Objective 1: Co-Design of the Tool Interface

#### Overview

**Figure 2:**
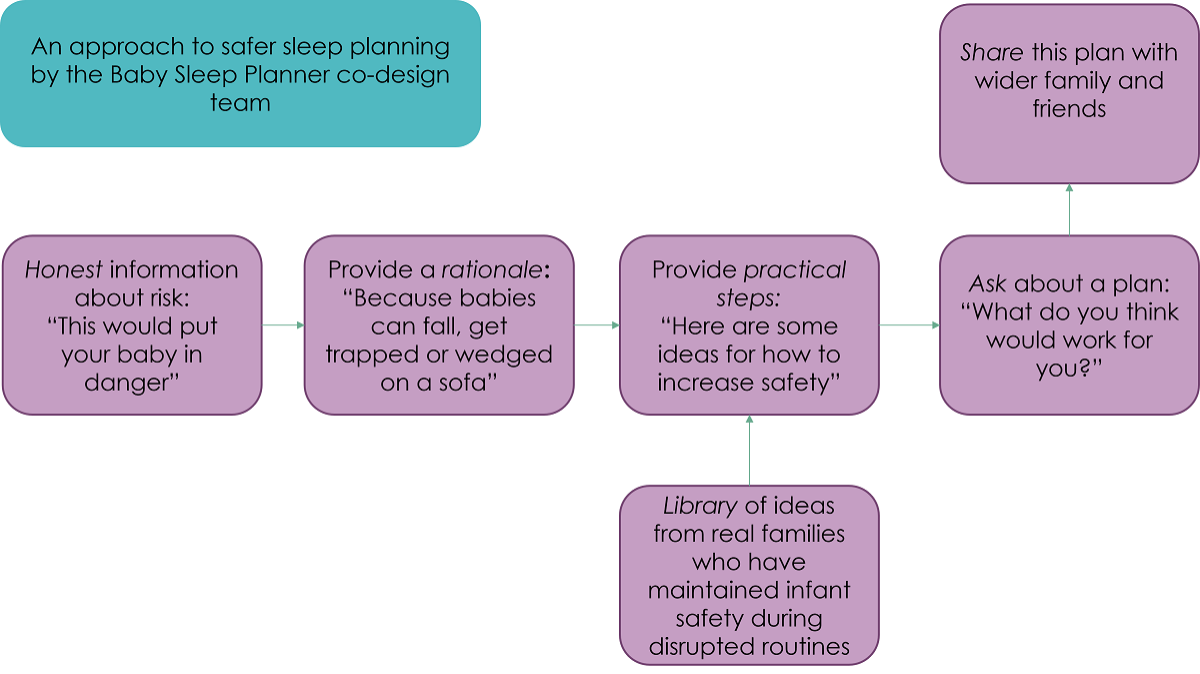
Risk communication model designed by the co-design team.

Our family advisory group, together with the research team, developed a model for delivering risk information (Figure 2) to caregivers of infants involving five stages: (1) being honest about the risks, (2) giving reasons for the risks and feedback on reducing them, (3) showing options for reducing the risks (using other families’ real experiences), (4) asking what would work and support planning, and (5) making it shareable for other caregivers. Using this input, we worked together with the software development team and a graphic designer to make the tool meet each of those 5 stages. We adapted the planning option wording and images based on the recommendations of the family advisory group and included advice specific to a wider range of families thanks to their focus on the realities of infant care, such as nonstandard housing and looking after more than 1 baby at a time.

Following the co-design meetings, we produced a flowchart of tool functions showing the questions and functions for each stage. This flowchart was refined through further family advisory group meetings and with feedback from our professional advisory group members. Decisions were made based on the complexity of the tool, the costs of the design, and how well it enabled each of the behavior change techniques.

During this process, we kept the risk assessment and sleep environment planning sections separate, with an option to complete them together if suitable. Feedback from professionals in our advisory group suggested that the separate risk assessment could be a useful stand-alone tool for professionals working with families to know who to target with additional support for safer sleep and to complete before using the tool with a family. The risk assessment is based on nonmodifiable family background and birth characteristics, whereas the sleep environment section is based on modifiable behavior.

An initial prototype of the tool interface was available for feedback from our professional advisors, after which any final refinements were made. Table 2 shows example changes made throughout the co-design process. The family and professional advisory groups also reviewed the training materials.

**Table 2:** Example changes from the co-design process and professional advisory groups.

Feedback, question, or wording in tool	Change	Reason and example
“Which one best describes your relationship to the baby?”	Add in an answer option for “both parents or caregivers”	Feedback from health professional advisor—if they are talking to both parents at the same time (eg, in midwifery clinic)
“Babies in larger families, especially if the mother is young, are nearly three times more at risk of SIDS.”	Change to the following: “Babies in families with 2 or more children, especially if the mother is young, are nearly three times more at risk of SIDS.”	Feedback from professionals and family advisors that 2 children is not really a “large” family
Feedback that the nonmodifiable nature of the risks feels unfair	Add in the following: “Your baby’s background risk is fixed and often not something you can control. You can have control over your baby’s sleep environment and reduce their risk greatly by following the advice.”	Feedback from family members; this change may help give families a feeling of acknowledgment that their baby’s risk status is not within their control and empower them to reduce risks by following safer sleep advice
Things to think about if answered “sheets or blankets”: “Make sure sheets and blankets can’t cover the baby’s face. Putting the baby at the bottom of the space can stop them wriggling under blankets.”	Change to the following: “Make sure sheets and blankets can’t cover the baby’s face. If baby is in a cot, putting their feet at the bottom of the cot can stop them wriggling under blankets.”	Feedback from family advisors so that people do not interpret “bottom of the space” as the bottom of an adult bed
Question: what will be covering the baby?	Add in options for “nothing.” If “nothing” is selected, add the following text: “If it is very hot in the room where the baby will sleep, it may be best not to use any bedding. You can also try to cool the room, please visit this site for more advice: [link to relevant Lullaby Trust web page]”	Feedback from health professionals and families during a heat wave to accommodate hot weather scenarios
Add in more detail when “blankets” is chosen: “Make sure sheets and blankets can’t cover the baby’s face. If baby is in a cot, putting their feet at the bottom of the cot can stop them wriggling under blankets.”	Change to the following: “Make sure sheets and blankets can’t cover the baby’s face. If baby is in a cot, putting their feet at the bottom of the cot can stop them wriggling under blankets. Make sure sheets and blankets don’t make the baby too hot—for advice about temperature please visit: [link to relevant Lullaby Trust web page]”	Feedback from health professional advisors to add information about thermal regulation and room temperature
Details about the risks of smoking	Added in a link to relevant Lullaby Trust web page.	Requested by professionals to support conversations about smoking cessation

#### The Baby Sleep Planner Intervention

**Figure 3:**
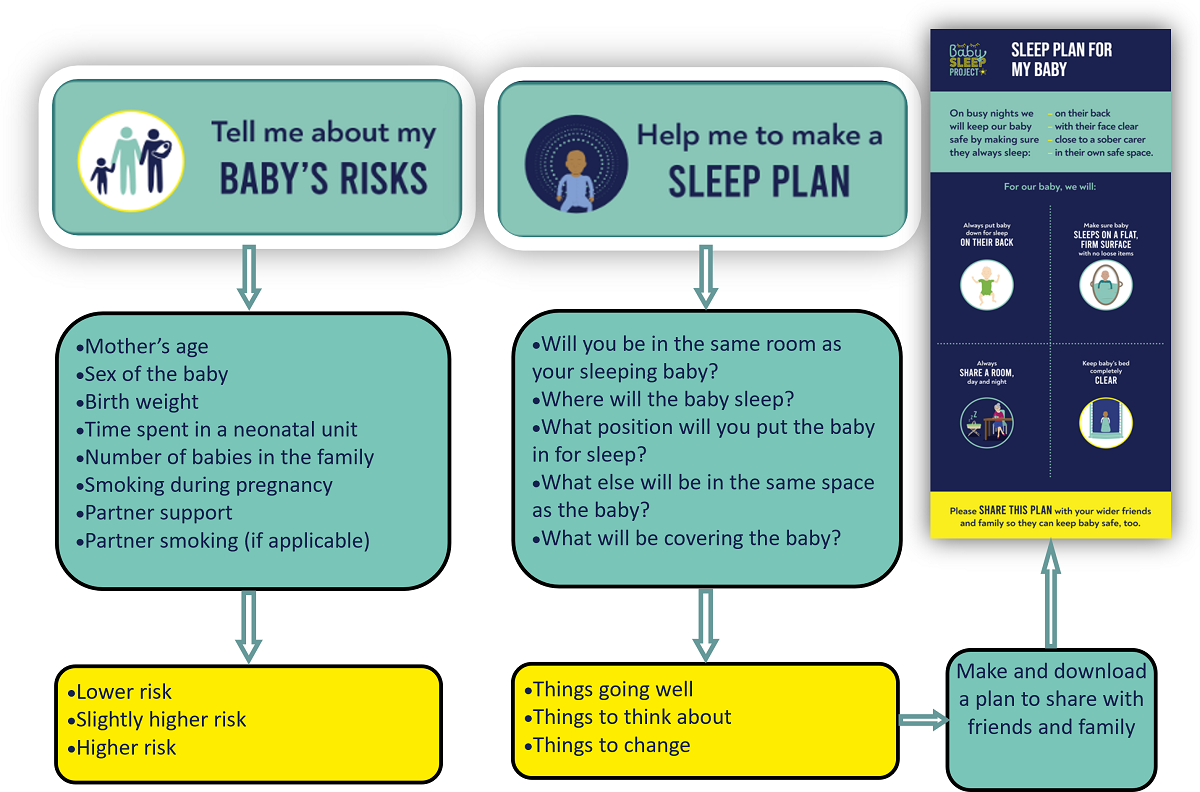
Final design of the Baby Sleep Planner.

The Baby Sleep Planner (Figure 3) is a web-based risk assessment and planning tool with 2 sections that can be completed together or separately. The risk assessment tool includes 8 questions about the background characteristics of the infant, usually delivered shortly after birth, and assigns a score based on an algorithm [14]. These questions include maternal age, number of children, smoking during pregnancy, partner support, partner smoking, infant sex, birth weight, and neonatal unit admission. A total of 7 other nonmandatory questions are asked to inform the research, including infant age, gestation, multiple births, ethnicity, relationship to the baby, whether this is the first time using the tool, and whether there is a health professional present. Each question is categorical, with 3 levels of risk assigned: lower, slightly higher, and higher risk. The information in the results is tailored to the risks present in each infant. The results are presented with information about research evidence and a key message that risks can be substantially lowered by following safer sleep advice.

The planning tool includes 6 questions about the infant’s sleep environment, including room sharing, sleep location (eg, cot or adult bed), sleeping position, items in the sleep space, coverings (eg, blankets or sleeping bag), and feeding method. The results of these questions comprise 3 categories: things going well, things to think about, and things to change. Feedback includes links to further information from a national advice charity, the Lullaby Trust. Users are then given 14 “plan options” comprising images with safety messages and asked to pick between 2 and 4 to create their own baby’s plan. The plan can be downloaded to a device (eg, mobile phone) as an image that can be shared with wider family and friends.

The intervention includes training for health professionals, a 30-minute web-based session with a member of the research team to explain the background, theory, and use. For this evaluation, the tool was only available for use as part of a conversation with a health professional, and the link to the website was not shared directly with families. Although the tool is under development, we wanted to make sure that the content and interpretations were as intended.

### Objective 2: User Testing, Including Tool Use Database Development

The tool and associated training were completed by 22 health professionals: 9 (41%) midwives, 8 (36%) health visitors, and 5 (23%) family nurses. In total, health professionals reported using the tool with 58 families, and the tool database recorded 55 uses of the tool. Of these responses, 48 (87%) were for the combined risk assessment and planning tool, 5% (3/55) were for the risk assessment tool only, and 7% (4/55) were for the sleep planner only. It was not possible to match tool use to a particular user because of data security, so we do not know whether the database responses are from real conversations with families or health professionals trying out the tool. We also do not know whether the tool was completed more than once per participant, although the reports from the health professionals suggest that it was not completed more than once. Health professionals reported that all the families they spoke to consented to seeing the tool, and the majority also consented to the research team seeing their answers. However, as we were unable to match the database responses, we could not quantify how many refused to provide information to the researchers, although it is thought to be a small number.

### Objective 3: Qualitative Interviews

#### Health Professional Interviews

A total of 22 health professionals volunteered to take part in the process evaluation (Table 3), were recruited for the study, and attended either over the web or face-to-face training. In total, 9% (2/22) of the health professionals subsequently withdrew, both going on long-term sick leave. In total, 20 health professionals, comprising 9 midwives, 8 health visitors, and 3 family nurses, were interviewed.

**Table 3:** Description of health professionals recruited for the process evaluation.

ID	Role (n=22)	Training in person or over the web (n=11 in person and n=11 over the web)	Families shown the tool (n=58), n (%)	Took part in an interview? (n=20)
MW1	Midwife	In person	2 (3)	Yes
MW2	Midwife	In person	5 (9)	Yes
HV1	Health visitor	Over the web	4 (7)	Yes
HV2	Health visitor	Over the web	3 (5)	Yes
HV3	Health visitor	Over the web	1 (2)	Yes
HV4	Health visitor	Over the web	4 (7)	Yes
HV5	Health visitor	Over the web	5 (9)	Yes
FNP1	Family nurse	In person	1 (2)	Yes
FNP2	Family nurse	In person	0 (0)	No—off sick
FNP3	Family nurse	In person	0 (0)	No—off sick
FNP4	Family nurse	In person	2 (3)	Yes
MW3	Midwife	In person	0 (0)	Yes
MW4	Midwife	In person	5 (9)	Yes
HV6	Health visitor	Over the web	5 (9)	Yes
HV7	Health visitor	Over the web	5 (9)	Yes
HV8	Health visitor	Over the web	6 (10)	Yes
MW5	Midwife	In person	1 (2)	Yes
MW6	Midwife	In person	4 (7)	Yes
MW7	Midwife	Over the web	1 (2)	Yes
MW8	Midwife	In person	0 (0)	Yes
MW9	Midwife	Over the web	3 (5)	Yes
FNP5	Family nurse	Over the web	1 (2)	Yes

#### Practical Use and Engagement

Health professionals found the tool easy to use and appreciated its ability to engage parents in conversations regarding the risk of SUDI. They commented on its simplicity, plain language, and visual design. Some commented that it took a little bit of time to get used to using it, and some had difficulties accessing the internet while with the families:

...it was really good, and parents really engaged with it, because it was very much tailored to them, and so compared to other sleep conversations they were much more engaged and interested in it.

HV7

With anything it takes a little bit of getting used to, but it was easy enough once you have done it once with a family. You were both learning at the same time really with the first family I did it with.

HV1

#### Communicating About Risk

Health professionals appreciated the balance between being honest and up-front with families while being careful to avoid making parents feel anxious or judged. They described using the tool to support conversations that empower parents with knowledge about their individual infants, which then supports the need for the safer sleep messages:

What I liked about the tool is that directness about it...it gave me support, because often I feel like as a practitioner I was saying this stuff and can come across a little bit naggy...Whereas this was a really helpful tool to back up what we’re asking of families.

FNP5

...you need to understand a certain level of risk, but it wasn’t making people feel worse going through the questions, and going through the outcomes, it didn’t make parents feel more anxious about the situation.

HV6

#### Beyond the Messages

Advice given to families is often didactic, and health professionals commented that the tool allowed them to go further than just giving out the safer sleep messages. They liked that it supported a conversation rather than just telling families what to do. They described being able to get more information across, feeling that parents were more involved in the conversation, and being able to delve into specific messages that parents wanted to discuss:

...she has some learning difficulties as well, so I was quite surprised she could focus throughout the whole thing really. It felt like that they were being involved, rather than just being told, and also I think what it did was it meant we talked about it for longer.

FNP1

...she could then ask questions just about little other areas, just talking about when can they have a pillow, what age? And it facilitated a little bit more of a wider discussion around safe sleep really.

FNP5

So engaging with people, listening to what they have to say, and then maybe just bringing it up more in conversation than this is what I’m telling you to do.

MW9

#### Wider Family Support

Advice about safer sleep is often given solely to the primary carer of the infant, and some health professionals described how they used the tool to encourage mothers to share their plans with their wider friends and family. Some found it difficult to send the image to the mother’s device and would have preferred a printed option; however, there was a consensus that supporting how mothers communicate about safer sleep with their families is important:

We used it to help her communicate what was important to her with the paternal family, so that she could ensure her baby was safe, and those steps that she was taking at home could be continued in a different environment.

FNP5

We find this with a lot of our clients’ parents in that they’re giving a lot of advice themselves, so it’s important that they are given the up to date advice so they can support the mum in making decisions.

FNP1

#### Barriers to Use and Changes Suggested

Health professionals cited time as a limiting factor in tool use as well as internet access problems and battery issues with work laptops. The timing of use was also raised, with some seeing value in repeating the process and starting sleep planning conversations during the antenatal period. They described seeing the value of the tool for all parents, not just those with infants at increased risk. Some suggested a variety of options for sharing the sleep plan image with the family, including printing it off for those without mobile devices. Changes suggested included versions in languages other than English, more information on the risks associated with smoking, rethinking our description of larger families, information on ideal room temperature, and more details on blanket use:

For some people having a visual maybe on the fridge printed off, that’s what I intended to do but it got lost in the ether when I downloaded it on my phone and I couldn’t find where it had downloaded to. For others, their phones break every week, and getting new numbers, something printed like that would be ideal.

MW1

More about smoking around the baby or smoking in the same room as baby?

HV2

Would you need a different tool for antenatals to look at the risk factors, and you could discuss those risk factors then with them?

HV4

The girl had only had two children, but it came up with a message at that point from babies from larger families are more at risk, and I wouldn’t class two children as a larger family.

MW3

#### Scope for Future Work

Health professionals were asked about other topics they thought would be of benefit to the families they worked with, and they raised a variety of issues. The limited capacity for home visits and relationship building owing to heavy workloads was a constant problem. Some had ideas for widening the scope of the Baby Sleep Planner to be able to use it during pregnancy and with non-English speakers, and 1 health visitor suggested adding reminders that could be sent via email to parents to support the changing needs of infants over the first year, for example, at 6 months, when babies can potentially be moved into a room of their own. Several suggested including more detail in the existing tool focusing on use of substances, both prescribed and illicit, to increase awareness of the risks associated with cosleeping when parental responses may be impaired. Another health professional suggested incorporating a planning aspect into stressful parenting situations, for example, planning activities to reduce stress and potential injury to the infant, similar to ICON (a program to reduce abusive head trauma in infants). Finally, 1 family nurse suggested an intervention focused on domestic abuse, in particular on the effects of coercive control on parenting capacity:

Something about domestic abuse? Domestic abuse like to care and control, and neglect, of them not being able to focus and care for their babies, because of stuff going on in their relationship.

FNP1

More information about smoking or strong painkilling medication that might make someone sleepy.

MW3

In the future would it send parents reminders and things at all if they signed up for this planner and things? I think that would be good. As health professionals we don’t see them as often as we can do, but just if they signed up they could get a text or whatever about sleeping, or if it was an app you would get a notification wouldn’t you about remember these things, it’s really important, your baby is 6 months, they can move into their own room, but they still can’t have a pillow or duvet, that kind of thing.

HV1

#### Family Member Interviews

Health professionals sent contact details for 32 family members who had agreed to be contacted about a research interview. All were invited to take part in an interview except for 1, whose contact details were sent to the research team after data collection had been completed and recruitment was closed. A total of 20 families gave consent to be interviewed and completed a telephone interview (Table 4). In total, 4 interviews included both the mother and the mother’s partner. Joint interviews were analyzed together, and 1 mother was still pregnant at the time of the interview. Risk scores (using the algorithm for interview participants’ infants) ranged from 0 to 153 (mean 58.7, SD 49.5). A total of 3 of the infants had risk scores of >115, indicating increased risk of sudden infant death syndrome using our recently developed algorithm.

**Table 4:** Families interviewed, with corresponding infant risk status.

ID	Relationship to baby	Maternal age (y)	Infant sex	Birth weight (g)	NICU^a^ admission	Parity	Smoking during pregnancy	Partner support	Partner smoking	Infant risk assessment score
01	Mother	<21	Male	≥2500	No	1	No	Yes	No	79
02	Mother	21-24	Female	≥2500	No	1	No	Yes	Yes	65
03	Mother	≥25	Male	≥2500	No	1	No	Yes	No	15
04	Mother	≥25	Female	≥2500	No	1	No	No	N/A^b^	18
05	Mother and partner	21-24	Male	≥2500	No	1	No	Yes	No	46
06	Mother	≥25	Male	≥2500	No	1	No	Yes	No	15
07	Mother	<21	Female	≥2500	No	1	Yes	Yes	Yes	146
08	Mother and partner	≥25	Male	≥2500	No	1	No	Yes	No	15
09	Mother and partner	≥25	Male	≥2500	No	1	No	Yes	No	15
10	Mother	<21	Male	≥2500	No	2	No	Yes	No	111
11	Mother and partner	≥25	Male	Pregnant	Pregnant	1	No	Yes	No	15
12	Mother	≥25	Male	≥2500	No	1	No	No	N/A	33
13	Mother	<21	Male	≥2500	No	1	No	Yes	No	79
14	Mother	≥25	Male	≥2500	No	1	No	Yes	No	15
15	Mother	≥25	Female	≥2500	Yes	2	Yes	No	N/A	114
16	Mother	≥25	Female	≥2500	No	1	No	Yes	No	0
17	Mother	≥25	Female	≥2500	No	2	No	Yes	No	32
18	Mother	<21	Female	1749-2499	No	1	Yes	Yes	Yes	153
19	Mother	<21	Male	≥2500	No	1	No	Yes	No	79
20	Mother	21-24	Female	≥2500	No	2	Yes	No	N/A	129

^a^NICU: neonatal intensive care unit.

^b^N/A: not applicable.

#### Tool as a Trusted Source

Parents commented on how they felt that they could trust the information they received from the tool and that this was supported by its delivery from a health professional and alignment with national advice. They appreciated the wording as “factual” and not judgmental. Some liked that it was interactive and tailored to their baby, whereas others felt that they knew the information already and this was just a useful reminder:

I thought it was presented very simply, but not in a patronising way. It just the imagery and the just having a few words around it just made sense, and made it a bit more engaging.

ID04

...it’s not judgemental but straight down the middle factual, but not trying to ward people off. I think the wording was fine for me.

ID03

...as a first time mum it was very useful, because I wouldn’t have...people tell me stuff, but to hear it from somebody professional who actually knows was a lot more helpful.

ID02

The hospital went over it when I was discharged, and my community midwife, but that was about it...They were the same but they weren’t in as much detail as your survey.

ID07

I think it’s good when they come across the whole planner about it, because I think a lot of people would like to go through it just so that they’ve got all the information they need as well.

ID20

#### Risk Assessment Process

Lower-risk parents reported feeling reassured by the results of the risk assessment, whereas higher-risk parents described it as unsurprising and supportive in that it encouraged them to follow the advice. Some described difficult feelings regarding the algorithm risks being unchangeable or related to things that they did not have any control over:

So yeah it was reassuring to know that as far as anyone can predict we are at lower risk. So that I found quite helpful.

ID04

I believe it came out that I was high risk, that it was high risk, but with doing everything that I’m doing she said it was okay, do you know what I mean?

ID15

The difficult one about with the single parent is unsupported partner. That’s the one thing that was difficult for me was you’re three times higher risk with SIDS, what can I really do about that? That was difficult. Tilted cot, fine I can change that, but I can’t change a supportive partner thing.

ID12

Yeah, and I think at the end when you get your risk as well and it’s like you’re at this much of a higher risk, it opens your eyes and you’re like wow and you’re like okay, do you know like...yeah.

ID20

#### Sleep Planning Process

Parents had mostly positive things to say about the sleep planning process, commenting that it included all the information they would need and appreciating that it explained the reasons for the advice without just telling them what to do. Most of the parents had answered the sleep environment questions remembering a real sleep that had taken place recently or with what they normally did. Changes to this part of the tool may be required to encourage parents and caregivers to use the sleep planning process to imagine what might happen in times when the normal routine is disrupted, for example, when staying away from home overnight. Several of the parents commented that they did not receive their plan image from their health professional:

Because as well the idea is that it doesn’t just tell you what to do or what you should do, it tries to explain why.

ID15

I think we’ve been quite realistic with our plan, so I think we could stick to it most nights, depending on how things go with the baby, things could change in terms of feeding patterns and that kind of thing. But I generally it would be quite straightforward to stick to.

ID03

One mother shared how she had used the sleep planning tool and downloaded the plan as a picture to share with her family members who were responsible for her baby’s overnight care once a week:

This is what we done, we took a picture so then we could send it to them, because I thought it would be more helpful to them, whereas if they don’t have him as much, so they’re not...they don’t know him as well in his sleep than what I would do. So I thought it would help them a lot more.

ID01

#### Changes Since Using the Tool: Potential Impact

Several parents described things that had changed as a result of using the tool with their health professionals. These changes included, taking items out of the Moses basket, tucking blankets in, using age-appropriate sleeping bags, and keeping unsupervised pets away from sleeping babies. Others felt that they were doing everything they could but still appreciated the reassurance that this gave them:

...it was nice that there was something on there that I hadn’t considered. I felt a little bit nervous about the fact that he’s been sleeping with a slant, but it was only for seven to ten days, and I’ve rectified that now.

ID12

I didn’t know that you didn’t have to...you weren’t allowed anything in the Moses basket.

ID07

...a lot of things we were doing already, and it was good to get the advice about tucking the blanket under the mattress, because that bit we had been like oh how do we keep it secure so it doesn’t go over his face? So yeah, no it was useful.

ID09

#### Barriers to Use and Changes Suggested

Some of the barriers included not being able to access the plan images and preferring to go through the tool on their own without their health professional present. Some suggested having more links to click through for more in-depth explanations of how the messages protect infants. Several parents felt that the way in which the risks were explained could be better, using pictures or comparators that were known to them. One parent commented that there could be more emphasis on the ways in which they can lower the risks and less on things that they cannot change:

...perhaps being able to click on something and go through to a bit more information. So as you were saying about the feet to foot of the bed, so if you want to know more about it you can click on the icon and go through and have a bit more information about why that’s the recommended sleeping position and those sorts of things.

ID04

I suppose the only thing that would be easier to use would be something digitally, like an app, or something that could be sent through to you to do rather than being shown two you on another laptop.

ID11

...having this baby here that I need to look after on my own all night with no support from his dad, and then to look at that statistic it was like oh no, and what can I do about that apart from bring down all the other risks? That’s the only feedback I would have in terms of there was no okay well what can I do to make sure that I’m lowering that risk in that way.

ID12

## Discussion

### Principal Findings

This study developed a risk assessment and planning tool that is pragmatic for use in a real-world setting. It has the potential to be used in clinical practice for the identification and support of high-risk infants and for families to use to reduce proven risk factors in the infant sleep environment. Interviews with users demonstrated how the tool could enable enhanced support to reach those most at risk while also reducing the burden of work for health professionals. Health professionals reported that they found the tool more conversational and less didactic, and families reported that they appreciated this approach.

### Comparisons With Existing Literature

Our findings align with those of other research into behavior change for this group, including a recent COM-B analysis of interventions to improve the uptake of safer sleep that found that, although increasing capability by passing on information about risks was common, more effective interventions incorporated motivational factors such as goal setting and making plans [11]. The risk assessment and planning tool incorporates motivational factors within the planning part of the tool, asking about where and how the baby will sleep, providing feedback on a variety of answers, and inviting users to prioritize their goals for safety in an individualized safety plan. In our study, families appreciated the approach to bed sharing taken in the tool, aligning with recently updated advice from the National Health Service in England to acknowledge that bed sharing occurs in planned and unplanned ways and offering advice to reduce the risks in bed-sharing situations [15]. In a 2016 review of behavioral interventions, Moon et al [16] suggested that interventions should be multilevel, incorporating contextual factors into the design, as we have attempted to do in this study. They also recommended formal process evaluations and future studies that can measure effectiveness as needed to support wider implementation [16]. Other reviews have found similar issues with measuring effectiveness and concluded that creative methods may be needed, as well as interventions that include the wider family and peers [17].

### Strengths and Limitations

The inclusion of a theory-based approach incorporating co-design elements, along with the evidence for behavioral influences that work for this group, gives this intervention a solid basis for effectiveness. Findings from the interviews support the theory that sharing individual risk status information (ie, “information about health consequences”) may increase parental understanding of their own infant’s safety needs. Sharing achievable and realistic plans may increase social support for following safer sleep advice, and having personalized conversations about safer sleep with health professionals as credible and trusted sources may enhance parental confidence and decision-making, especially during times of disruption to the normal routine. Integrating feedback from both health professionals and family members into the design and function of the tool meant that we were able to align the needs of both groups by ensuring that the tool provides evidence-based information in a way that supports the individual needs of each family. Testing the prototype intervention under real-world conditions provided insights into implementation and highlighted necessary changes. Issues with accessing the downloaded plan image meant that some families were not able to use this aspect of the tool, and this needs further consideration, with options for printing where possible. There were promising signals from the evaluation that understanding the risks to their baby and planning for safety during times of disruption may influence decision-making regarding the sleep environment, prioritizing safety at all times. The finding that some families found the unchangeable risks difficult to hear prompted further work to investigate how the risk assessment results can be presented as honest but not hopeless, providing more emphasis on how safer sleep planning can reduce the risks as much as possible even for an already higher-risk infant. It may be that the decision to describe infants as “higher risk” is unhelpful, and this should be explored in future work. This wording is currently used based on advice from our family advisors that being honest about the risk status of infants is important, as shown in the model in Figure 2. Most of the suggestions for changes to the tool were mainly minor, and we were able to incorporate them fairly quickly (see Table 2 for examples). Other changes, such as non–English language versions, will take longer. This was a small evaluation study using a prototype intervention. Our original aim included the development of a stand-alone tool that families could use independently, but studies that can collect more data on the safety and appropriateness of the tool to be used in this way will be required first. The risk status of the infants referred to the research team was also lower than we had expected; only 15% (3/20) of the families had infants at increased risk of SUDI, although every family except 1 had at least 1 risk factor present. We were also only able to include English-speaking families in this study, and future work to translate the tool for use in other languages should be included as part of future evaluations. Challenges with health professional recruitment because of current National Health Service pressures led to delays in data collection, resulting in health professionals using the tool with any families they thought suitable rather than those with higher-risk infants only. We were also unable to analyze the background tool data in this study as we were not able to discern “real” conversations with families from health professionals practicing with the tool. Improvements to tool background data collection have been made to make it possible to use these data to understand the characteristics of the families using the tool in future studies. We have also changed the wording of our “data usage” question to prevent this problem in future studies. To test the tool in “real-world” conditions, we did not restrict health professionals in terms of who they used the tool with, and some of them reported that they appreciated being trusted to use the tool with whom they thought best, including anxious parents with low-risk infants who would be reassured by the results. The implications of tool use for this population should be included in future evaluations without undermining the focus on families with infants at increased risk.

### Conclusions

The Baby Sleep Planner was designed with involvement from families and key stakeholders and shows promise as a useful tool for health professionals having conversations about safer sleep with caregivers of infants. The web-based tool was acceptable to family members, midwives, health visitors, and FNP nurses. Further work should investigate whether the uptake of this intervention will significantly reduce known risk factors in the infant sleeping environment associated with sudden infant deaths and whether this algorithm can identify families with infants at risk of other causes of death.

## Abbreviations

COM-B: Capability, Opportunity, and Motivation–Behavior
FNP: Family Nurse Partnership
SUDI: sudden unexpected death in infancy
UNICEF: United Nations International Children’s Emergency Fund

## Data Availability

The data sets generated during and analyzed during this study are available at the University of Bristol data repository, data.bris [18].
